# Mining of a Clinical Database: The Interpretation of Intense Serial Procalcitonin in the Prediction for Bloodstream Infection

**DOI:** 10.3389/fmed.2021.691793

**Published:** 2021-10-06

**Authors:** Zhiyi Jiang, Ning Liu, Luhao Wang, Jianfeng Wu, Xiangdong Guan

**Affiliations:** Department of Critical Care Medicine, The First Affiliated Hospital of Sun Yat-sen University, Guangzhou, China

**Keywords:** procalcitonin, bloodstream infection, bacteremia, fungemia, critical patients, sepsis

## Abstract

**Background:** Procalcitonin (PCT) is a promising biomarker for predicting infection. Bloodstream infection (BSI) is usually a deteriorating stage of sepsis. The purpose of this study was to explore the predictive value of intense serial PCT assays for BSI in the intensive care unit (ICU).

**Methods:** This study was a retrospective study based on a clinical database. We analyzed the data of critically ill patients from February 2016 to May 2020. The patients who received PCT assays and blood cultures (BCs) were classified into four groups according to the BCs: (i) BC negative, (ii) bacteria positive, (iii) fungi-positive, and (iv) combined-positive, and the patients with bacteremia were further subdivided into Gram+ and Gram– bacteremia.

**Results:** The database included 11,219 patients. There were 3,593 patients who met the criteria for the analysis. The PCT concentration differed significantly across BC groups (*p* < 0.0001). The fluctuation of PCT significantly increased in the BC positive groups (*p* < 0.0001). According to the receiver operating characteristic (ROC), the optimum cutoff of the fluctuation of PCT was around 8 ng/ml for predicting BSI.

**Conclusion:** Our study indicated that the fluctuation of PCT could be an indicator for screening BSI, but less accurate for Gram-positive infections. With a fluctuation of PCT less than 8 ng/ml, BSI should not be a rational cause for sepsis exacerbating.

## Background

The new definition of sepsis had changed the diagnosis criteria of the severe general infection syndrome (1). As a result of the vague relationship between the sequential organ failure assessment (SOFA) score and sepsis, a specific and sensitive indicator for screening infection is still attracting the interest of clinicians. Bloodstream infection (BSI) is a distinctive cause of sepsis with greater in-hospital mortality (2). The gold standard for BSI diagnosis remains to be culture-based, though it is challenged with unsatisfactory sensitivity and poor timeliness in clinical practice (3–5). Despite next-generation sequencing (NGS) and the application of machine-learning methods have shown promising results in the diagnostic of BSI, it is not conventional and poor cost-effective at the present stage (6).

The available laboratory test for screening general infection includes C-reactive protein (CRP), white blood cell count (WBC), neutrophil-lymphocyte count ratio (NLCR), procalcitonin (PCT), and so forth (2). Over the last decades, those biomarkers are widely accepted as a screening system guiding clinicians to initiate the sepsis bundle timely. However, the predictive values of those biomarkers for critical infection varied in previous studies (3–5). PCT is a promising biomarker. Even though it was regarded as an indicator for de-escalation therapy of antimicrobial agents, the PCT-guided therapy showed no beneficial effect on mortality among septic patients (7, 8). Given that the former studies showed that PCT was unspecific to differentiate between sterile inflammation (SIRS) and sepsis, it is assumed that a single test for serum PCT might not be eligible for screening infection inflammation (9–11).

Based on intense serial detections, our study was conducted aiming at further exploring the diagnostic role of PCT for BSI.

## Methods

### Study Design and Settings

This study was a retrospective, single-center clinical study based on an electronic database from the Hospital Information System (HIS). The Information Data Centre and the Independent Ethics Committee (IEC) for clinical research and animal trials of the First Affiliated Hospital of Sun Yat-sen University (SYSU) approved the study protocol [IEC no. (2020) 266]. All the data in this study were retrieved from the records of the patients transferred into the intensive care unit (ICU) of the First Affiliated Hospital of SYSU. The ICU aforementioned is a tertiary care general ICU with above 2,000 admissions per year.

### Study Population and Definitions

The patients admitted to the ICU between February 2016 and May 2020 who received blood cultures (BCs) and PCT detections were included in this study. Under the daily assessment, the suspected patient had been screened by an attending intensivist according to both clinical symptoms and risk factors (2, 12). The therapeutic bundle was executed to all suspected patients in accordance with the guidelines, such as Surviving Sepsis Campaign for Sepsis, the Management of Candidiasis, and so forth (1, 2). Serial PCT detections had been requested to carry out daily to evaluate the course of antibiotic regimen ever since the patient was transferred to ICU (13, 14). Double sets of BCs (peripheral veins or arterial lines) were conducted in necessity. Those patients who had more than one BCs during the same ICU admission would be recorded. The population was classified into four groups according to the BC results: group1 for BC negative, group 2 for bacteria-positive, group 3 for fungi-positive, and group 4 for combined positive (Gram+ and Gram–, or bacteria and fungi). For further analysis, the patients in group bacteria-positive were subdivided into Gram-positive (group 2p) and Gram-negative (Group 2n) bacteremia.

### Data Collection

The following data were available in the electronic database: patient identification number, patient age at the time of blood collection, date and time of blood sampling for BC and PCT assay, BC result (positive or negative), microorganism genus and species in case of positive BC, and blood PCT concentration (ng/ml). Other demographic data, but no clinical data, were recorded. The frequency of PCT measurements, the minimum and maximum serum PCT concentrations would be retrieved from the serial detections of the same patient. Correspondingly, to display the fluctuation, the gap (PCT_max_-PCT_min_) and the ratio of PCT variations (PCT_gap_/PCT_min_) would be calculated according to the retrieved data. The data were combined into an electronic spreadsheet (Excel, Microsoft 365).

### Laboratory Information

The PCT concentration had been measured using an automated Elecsys assay (Roche cobas e 601, Elecsys BRAHMS PCT Roche Diagnostics GmbH, Germany). The normal PCT concentration was defined as <0.05 ng/ml. The double-set BCs had been obtained from different sites. Each set of BCs consisted of aerobic and anaerobic culture bottles. As soon as the bottles were transported to the laboratory, blood samples were loaded into the automated BC system (BACT/ALERT 3D, Biomérieux, France) and incubated for at least 5 days (no more than 7 days) or until the instrument indicated the culture was positive (15). Aliquots from positive bottles were subcultured on a blood agar plate for subsequent identification (35–37°C for 24 h).

### Aims

Primarily, the study was aimed to explore the diagnostic value of the intense serial PCT assays for screening BSI in critically ill patients. Secondarily, the study was aimed to look for the cutoff of the fluctuation of PCT for predicting or excluding BSI in critically ill patients.

### Statistical Analysis

All variables were tested for normality using the Kolmogorov-Smirnov test. Since none of the variables was normally distributed, they are presented as medians and interquartile range (IQR). According to the homogeneity test, the Kruskal-Wallis test was used to compare medians across the BC groups and for other comparisons of interest. Receiver operating characteristic (ROC) curves were constructed to evaluate the sensitivity and specificity of the PCT in predicting BSI. ROC curves displayed sensitivity vs. 1-specificity such that the area under the ROC curve (AUROC) varied from 0.5 to 1.0, with higher values indicating increased discriminatory ability. CIs on the AUROC were calculated using nonparametric assumptions. To identify differences between the AUC of individual ROC curves, the method described by Hanley and McNeil was used (16), with a *p* < 0.05 considered to represent a statistically significant difference. The optimum cutoff value was calculated based on the highest sensitivity and specificity combined (Youden index). Statistical analysis was performed using SPSS version 26 (SPSS Inc., Chicago, IL, USA).

## Results

### Patient Characteristics

**Figure 1:**
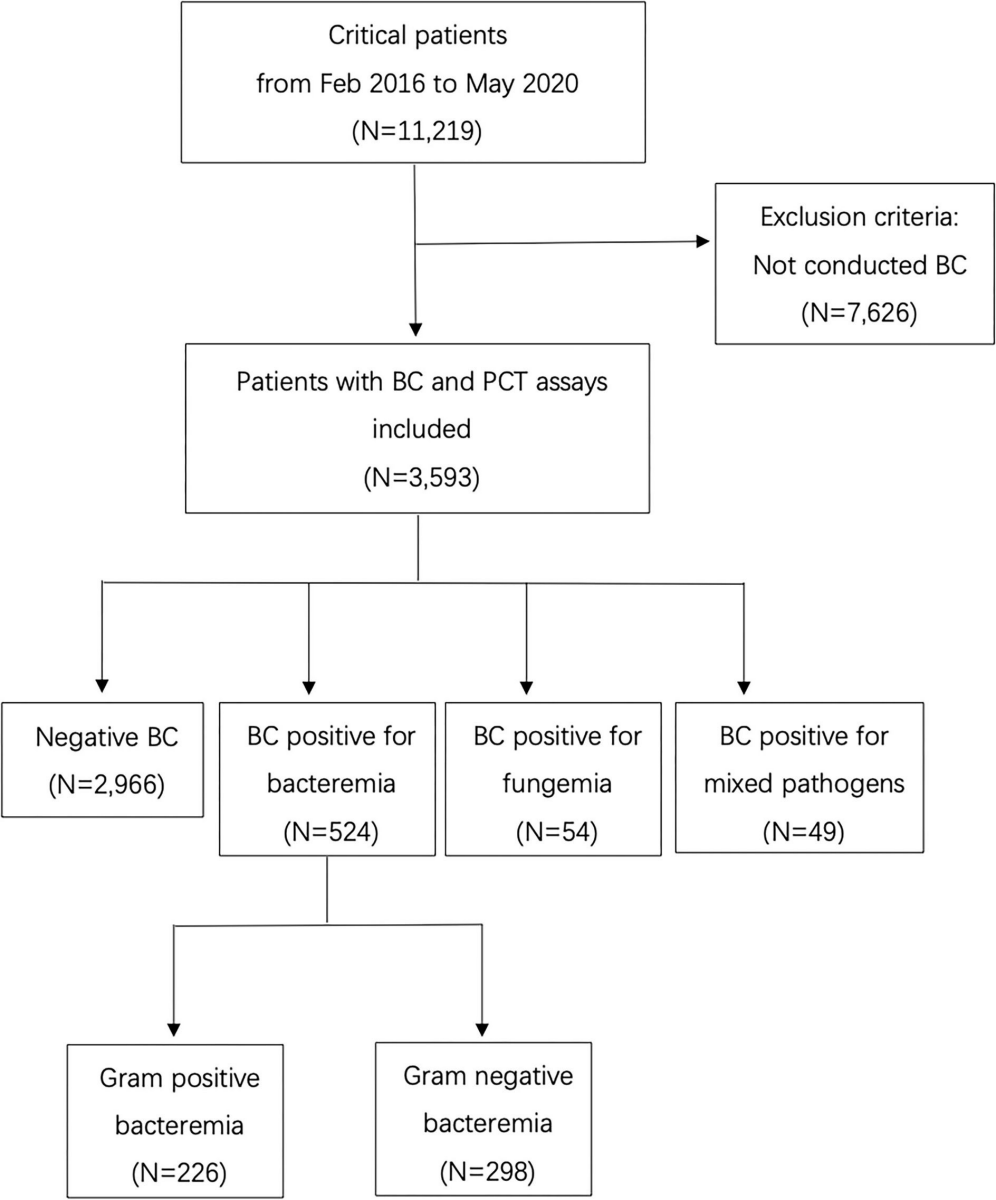
Flow chart of the inclusion and exclusion of patients (BC, Blood culture; PCT, Procalcitonin).

The database included 11,219 records of the patients. There were 3,593 patients who met the criteria for the analysis. A detailed flow chart is shown in Figure 1. The median age of the patients was 54 (41–65) years, 68.0% were men, 99.9% Asian. The median length of hospital stay (days) was 27 (16,41) in BC negative population and 28 (16,49) in BC positive population (*p* = 0.011).

### Blood Cultures

**Figure 2:**
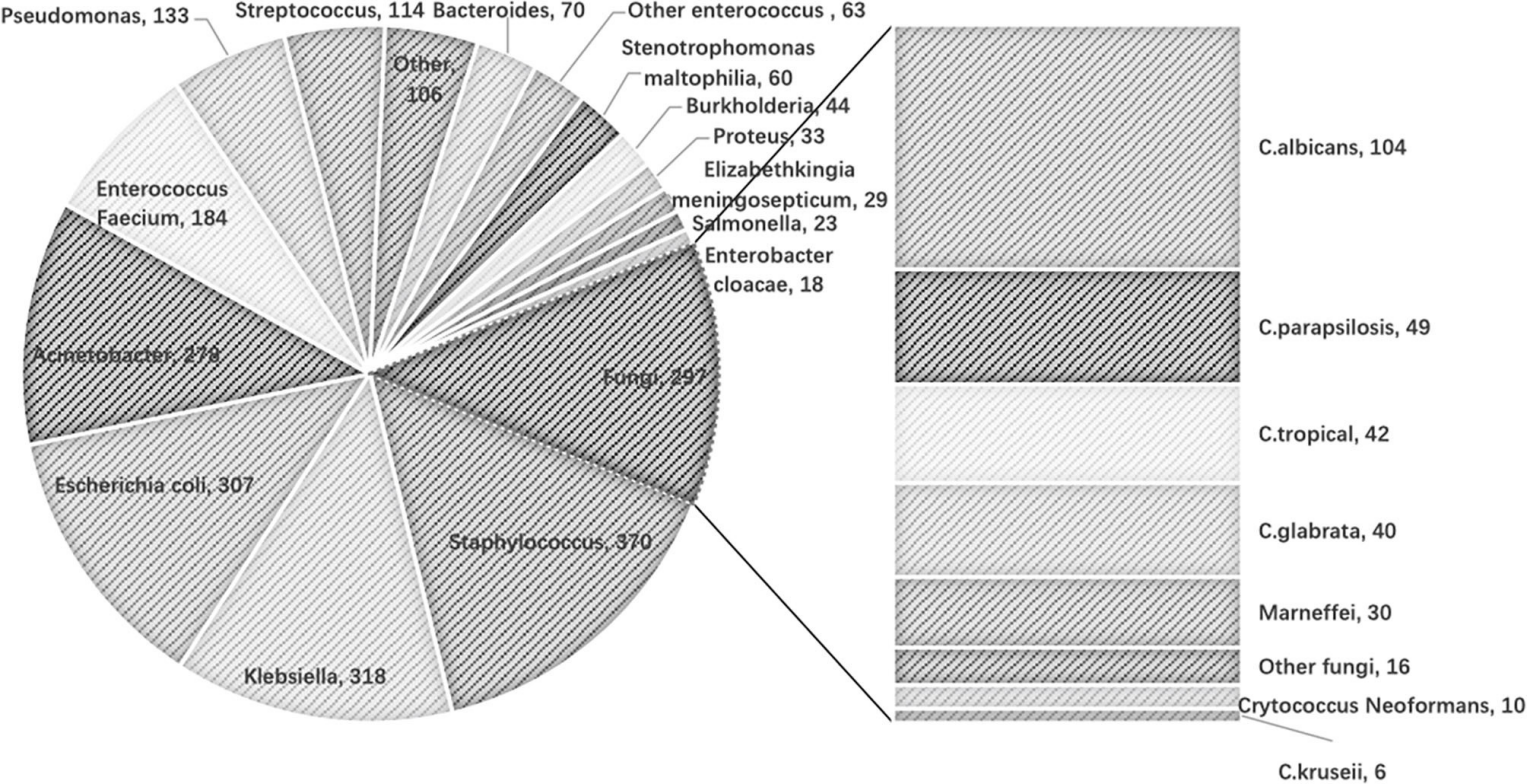
Distribution of the microbiological isolates in study cohort.

There was a total of 16,540 BC samples in the study cohort. The number of those positive samples was 1,471 (8.89%). Among those positive samples, 97 BCs were positive for more than one pathogen (five BCs positive for three pathogens). The distribution of the microbiological isolates is shown in Figure 2. Gram-negative bacteria were mostly cultured, followed by Gram-positive bacteria, then fungi.

The frequency of BCs in the study population was 4.6 times per patient on average. According to the microbiological isolates, the population was classified into four groups (Table 1). Positive blood culture was observed in 627 patients. There were 524 patients' BC positive for bacteremia (n_Gram+_ = 226, n_Gram−_ = 298), 54 patients' BC positive for fungemia and 49 patients' BC positive for combined pathogens (Table 1).

**Table 1:** The procalcitonin concentrations across the blood culture groups.

	**PCTmin**	**PCTmax**	**PCTgap**	**PCTratio**	**PCTfreq**
Group 1 *n* = 2966	0.15 (0.06, 0.49)	3.17 (0.67, 14.88)	2.68 (0.47, 13.5)	12.00 (4.00, 50.98)	9 (4, 16)
Group 2 *n* = 524	0.23 (0.08, 0.80)	11.14 (2.31, 52.38)	10.31 (1.69, 49.75)	28.91 (7.59, 131.98)	13 (6, 23)
Gram+ *n* = 226	0.15 (0.06, 0.64)	4.70 (0.97, 17.46)	3.99 (0.67, 15.31)	15.33 (4.91, 69.33)	11 (5, 21)
Gram– *n* = 298	0.29 (0.11, 0.95)	24.31 (6.11, 87.09)	23.15 (4.79, 80.03)	55.03 (10.89, 201,92)	14 (7, 26)
Group 3 *n* = 54	0.41 (0.18, 1.64)	26.09 (8.56, 86.06)	30.53 (5.07, 78.14)	56.88 (7.19, 165.68)	15 (8, 29)
Group 4 *n* = 49	0.36 (0.16, 1.91)	24.77 (14.51, 92.60)	24.63 (13.47, 92.44)	60.51 (13.21, 188.75)	16 (9, 29)

*Group 1: BC negative; Group 2: bacteria-positive; Group 3: fungi-positive; Group 4: combined positive*.

*Gram+: Gram stain positive; Gram–:Gram stain negative*.

*PCT, procalcitonin (ng/ml); PCTgap, PCTmax–PCTmin; PCTratio, PCTgap/PCTmin; PCTfreq, the frequency of PCT test in the records of each patient's records*.

### Procalcitonin

The median frequency for PCT assays was 9 times per patient in the BC negative group and at least 11 times per patient in the BC positive groups (Table 1). The PCT concentrations differed significantly across groups (Table 1, *p* < 0.001). The maximum difference in consecutive concentrations of PCT significantly increased in patients with microbiological isolated compared to those with negative BC (*p* < 0.001). The most obvious fluctuation of PCT was found in the fungemia group (PCT_max−min_ 30.53 ng/ml; IQR 5.07, 78.14), followed by the Gram-negative BC group (PCT_max−min_ 23.15 ng/ml; IQR 4.79, 80.03), and the least was in BC negative group (PCT_max−min_ 2.68 ng/ml; IQR 0.47, 13.5).

### Predictive Value

**Figure 3:**
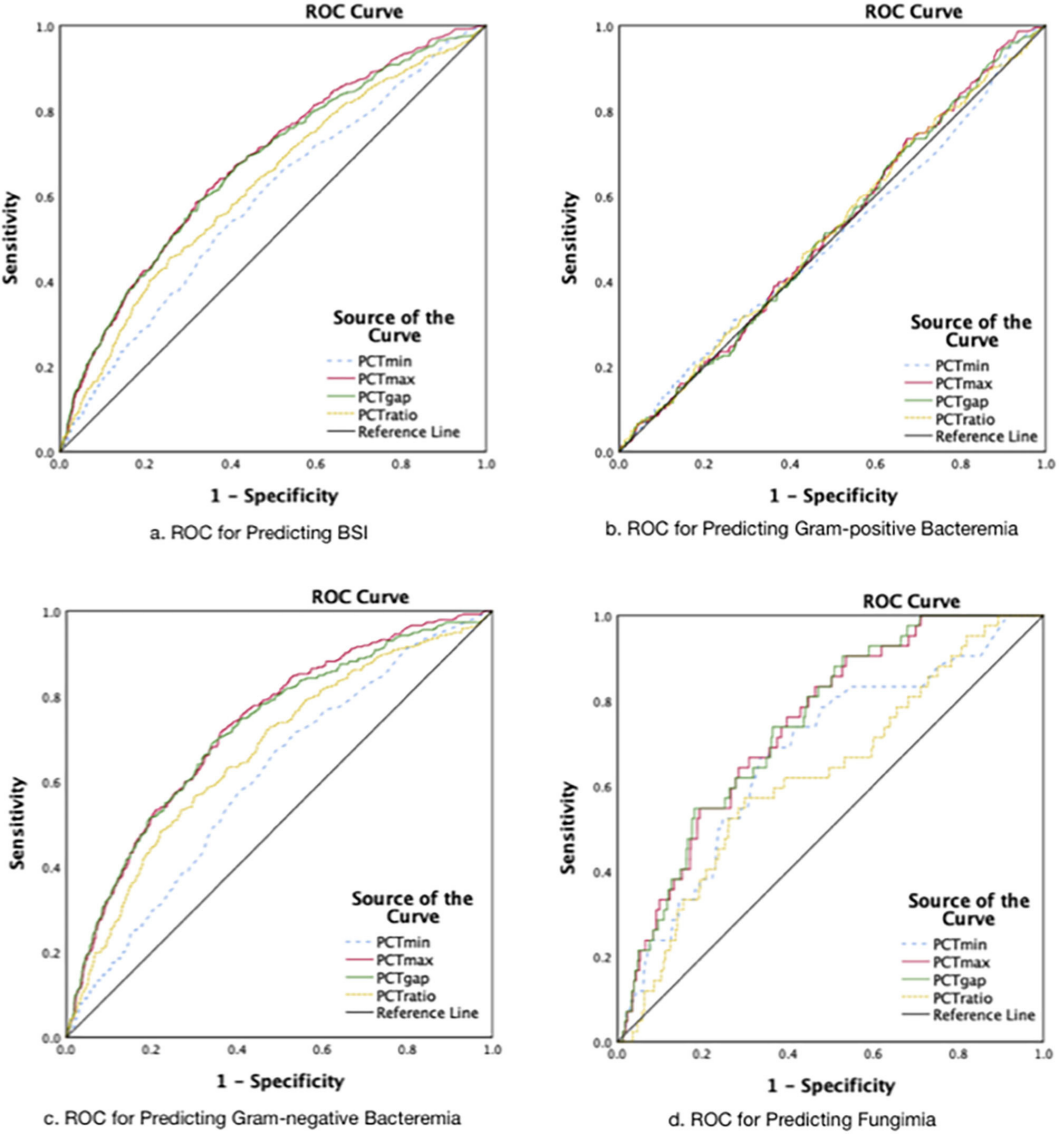
The receiver operating characteristic curves. **(A)** The AUROCs: for PCTmin 0.59.for PCTmax 0.68, for PCTgap 0.67, for PCTratio 0.62 (Group 2+3+4 vs. Group l; *P* < 0.001). **(B)** The AUROCs: for PCTmin 0.50 (*P* = 0.96), for PCTmax 0.52 (*P* = 0.41), for PCTgap 0.51(*P* = 0.50), for PCTratio 0.52 (*P* = 0.41). **(C)** The AUROCs: for PCTmin 0.61, for PCTmax 0.73, for PCTgap 0.72, for PCTratio 0.67 (*P* < 0.001). **(D)** The AUROCs: for PCTmin 0.68, for PCTmax 0.74, for PCTgap 0.75 for PCTratio 0.63 (*P* < 0.001).

According to the classifications of isolates, the ROCs are presented in Figure 3. The AUROC for the prediction of BSI was better for PCT_max_ and PCT_gap_. As a result, both of the parameters that quantified the fluctuation of PCT were selected to further investigate for cutoff values. The best cutoff values for the fluctuation in predicting BSI were around 8 ng/ml. The sensitivity, specificity, positive predictive value (PPV), and negative predictive value (NPV) are presented in Table 2. The combined-positive groups were not analyzed separately in this part.

**Table 2:** Receiver operating characteristic curve analysis to determine the optimum cutoff value of the PCT (ng/ml) for the prediction of BSI.

		**AUROC (95% CI)**	***p***	**Cutoff**	**Sensitivity**	**Specificity**	**PPV**	**NPV**
BSI	PCTmax	0.68 (0.65, 0.70)	<0.001	8.24	0.60	0.66	0.15	0.82
	PCTgap	0.67 (0.65, 0.69)	<0.001	8.10	0.57	0.67	0.25	0.89
Bacteremia (Gram–)	PCTmax	0.73 (0.70, 0.76)	<0.001	8.06	0.72	0.64	0.15	0.96
	PCTgap	0.72 (0.69, 0.75)	<0.001	8.08	0.69	0.66	0.16	0.96
Fungemia	PCTmax	0.74 (0.68, 0.81)	<0.001	12.70	0.72	0.69	0.04	0.99
	PCTgap	0.74 (0.68, 0.81)	<0.001	8.13	0.76	0.64	0.03	0.99

*Gram–, Gram stain negative; PCT, procalcitonin; PPV, positive predictive value, NPV, negative predictive value; BSI, bloodstream infection; AUROC, area under the ROC curve*.

## Discussion

The present study was based on big clinical data with intense serial PCT detections. With the consecutive PCT assays, we found that the fluctuation of PCT was an indicator for screening BSI and suspending antibacterial therapy. Because the negative predicting value was remarkably high, BSI should not be a rational cause for exacerbation in critically ill patients with a fluctuation of PCT less than 8 ng/ml. Meanwhile, the discriminating power of PCT for Gram-positive bacteremia was poor. It should be noticed that Staphylococcus, as a commonly potential contaminant, accounted for the largest proportion among the Gram-positive bacteria isolated in this study. Therefore, the predicting value of PCT for Gram-positive bacteremia should be interpreted with caution.

Previous studies had demonstrated the ability of serum PCT concentration in predicting bacteremia (5, 8, 17). In those studies, a single PCT assay was performed as a concomitant (within 6 h) assay with blood culture. The PCT was reported to peak at 16–24 h after commencing empirical antimicrobial therapy in suspected infection patients (18). Therefore, the single-test mode for PCT detection should not be recommended for septic patients. A study conducted on interval detections (0, 2, and day 4) failed to demonstrate the discriminating ability of PCT (19). Serial PCT evaluation was performed in a minority of clinical research (12, 20). In the Multicenter Procalcitonin Monitoring Sepsis (MOSES) study, the daily PCT assays over the first 5 days in septic patients were only reported as a significant prognostic indicator, but not a diagnostic biomarker (12). Furthermore, PCT might be peaked before or after the onset of BSI, consequently, the description of baseline level was vague. In our study, PCT assay was requested as a daily test throughout the antibiotic regimen. The baseline PCT of different individuals could be better estimated by the trough concentration. More importantly, the maximum difference among the serial PCT concentrations revealed the fluctuation of the biomarker through the course of BSI. As a supplement to the previous studies, within intense serial PCT tests, the fluctuation of PTC concentration developed its diagnostic role for BSI and expanded the indications for clinical application.

It was unusual that the PCT fluctuated significantly in the fungemia group. In a similar study focused on the concomitant PCT assay and blood culture, the author assumed that the PCT concentration at 10 ng/ml might help identify patients who will not benefit from empirical antifungal therapy pending blood culture results (16). Although PCT was reported as a specific indicator for non-virus infection diseases (10), there was no persuasive argument on differentiating invasive fungal infection (IFI) from other infections by PCT assay (21, 22). To explain the fluctuation in the fungemia group, a further investigation into the present database disclosed that approximately 80% of patients in the fungemia group (42/54) combined with focal bacterial infection, such as pneumonia, urinary infection, and intra-abdominal infection (the patients without focal bacterial infection in this group are listed in Supplementary Table 1). Hence, the fluctuation of PCT might be a preliminary indicator for screening fungemia in some undeveloped regions where the daily nonculture diagnostic tests for IFI were not available, but the aggravation of primary focal infection should always be a plausible explanation for the abnormal fluctuation of PCT.

The prevalence of BSI was reported ranging from 7.64 to 32.14% in the previous studies among suspected infection patients (23). In our study, based on that the positive rate of blood culture was 8.89% (1,471/16,540), the prevalence of BSI in the high-risk population was estimated to be 17.45%. The prevalence of BSI could explain why the PPV of PCT for screening BSI was merely 15–25% in our study and even poorer for fungemia with lower prevalence (24, 25). To further explore the predictive value of PCT for BSI, clinical-grade metagenomic testing should be included in future research (26, 27).

Even though the sample size is large enough in the present study, there are still several limitations. First, the study population is highly heterogeneous, which may affect the generalizability of the results, and the definition of PCT_gap_ is of limited clinical value. Second, the pathogens of infection focus are not fully investigated in this study. This might contribute to the slight fluctuation of PCT in the BC negative group. Third, the regimen of antibiotics, which were not contained within the present database, might conceal the actual prevalence of BSI (28). To further explore this issue, microbial cell-free DNA sequencing may be a crucial assay other than the antimicrobial regimen alone (14, 29).

## Conclusion

The present study indicates that intense serial PCT assay is essential in critical care, and the fluctuation of serum PCT concentration is a valuable indicator for screening BSI, but less accurate for Gram-positive infections. BSI should not be a rational cause for an exacerbating critical patient with fluctuation of PCT concentration less than 8 ng/ml. It is believed that our findings would contribute to screening the causes for aggravation during sepsis.

## Data Availability Statement

The raw data supporting the conclusions of this article will be made available by the authors, without undue reservation.

## Ethics Statement

The studies involving human participants were reviewed and approved by the Independent Ethics Committee (IEC) for clinical research and animal trials of the First Affiliated Hospital of Sun Yat-sen University. Written informed consent from the participants' legal guardian/next of kin was not required to participate in this study in accordance with the national legislation and the institutional requirements.

## Author Contributions

ZJ, NL, JW, and XG contributed to the study conception and design and drafted the manuscript. ZJ and LW contributed to the acquisition of data. ZJ and NL analyzed and interpreted the data. ZJ and NL are joint first authors. JW and XG are joint senior authors. All the authors revised the manuscript for important intellectual content, read and approved the final manuscript.

## Conflict of Interest

The authors declare that the research was conducted in the absence of any commercial or financial relationships that could be construed as a potential conflict of interest.

## Publisher's Note

All claims expressed in this article are solely those of the authors and do not necessarily represent those of their affiliated organizations, or those of the publisher, the editors and the reviewers. Any product that may be evaluated in this article, or claim that may be made by its manufacturer, is not guaranteed or endorsed by the publisher.
