# Supplementary material for: Mining of a Clinical Database: The Interpretation of Intense Serial Procalcitonin in the Prediction for Bloodstream Infection
Source: Front Med (Lausanne). 2021 Oct 6;8:691793. doi: 10.3389/fmed.2021.691793 (PMC8526537; doi:10.3389/fmed.2021.691793)
Supplement: Supplementary file 1 [file Table_1.DOCX]

Supplement

Table-1 Patients without focal bacterial infection in the Fungemia Group

| ID | Gender | Age (yrs) | PCTmin (ng/ml) | PCTmax (ng/ml) | PCTfreq | Species |
| --- | --- | --- | --- | --- | --- | --- |
| 01 | Male | 58 | 16.84 | 71.6 | 4 | C.albicans |
| 02 | Male | 73 | 0.04 | 8.28 | 16 | C.tropical |
| 03 | Female | 31 | 0.16 | 1.24 | 12 | C.albicans |
| 04 | Male | 71 | 0.61 | 40.94 | 6 | C.glabrata |
| 05 | Male | 33 | 0.34 | 137.8 | 28 | Marneffei |
| 06 | Male | 72 | 0.54 | 150 | 31 | C.parapsilosis |
| 07 | Male | 60 | 4.17 | 13.09 | 3 | C.glabrata |
| 08 | Male | 79 | 37.83 | 97 | 3 | C.albicans |
| 09 | Male | 3 | 0.59 | 233.5 | 5 | C.kruseii |
| 10 | Female | 63 | 6.61 | 76.77 | 15 | C.kruseii |
| 11 | Male | 62 | 1.45 | 9.58 | 20 | C.albicans；Trichosporon |
| 12 | Female | 60 | 0.23 | 15.92 | 12 | Trichosporon |
